# A Chromatic Enzymatic Time-Temperature Integrator Device Based on the Degradation of Phenolic Compounds for the Real-Time Prediction of the Quality and Shelf Life of Cherries

**DOI:** 10.3390/foods12061240

**Published:** 2023-03-14

**Authors:** Pedro D. Gaspar, Joel Alves, Adriana S. Quelhas, Christelle Domingos, Susana Caio

**Affiliations:** 1 Department of Electromechanical Engineering, Univerity of Beira Interior, Rua Marquês de D’Ávila e Bolama, 6201-001 Covilhã, Portugal; 2 C-MAST—Center for Mechanical and Aerospace Science and Technologies, Rua Marquês de D’Ávila e Bolama, 6201-001 Covilhã, Portugal; 3 InovCluster—Agroindustrial Cluster Association of the Center, Zona Industrial de Castelo Branco Rua A, 6000-459 Castelo Branco, Portugal

**Keywords:** cherry, TTI (time-temperature integrators), enzymatic TTI, fruit quality

## Abstract

The particular characteristics of cherries, such as color, firmness, and palate increase their demand, as does, among other things, their antioxidant properties that benefit human health. However, their high perishability leads to a reduced shelf life and consequently generates undesirable changes in the cherry flow chain. To ensure food quality and safety and prevent food waste, a smart device prototype is proposed. The concepts related to the formulation and design of the enzymatic-type chromatic time-temperature integrator (TTI) device used to monitor the real-time quality of cherries are described. The kinetic parameters for thermal inactivation of cultivar Santina cherries were determined based on the degradation of phenolic compounds that are substrates of the polyphenol oxidase enzyme, whose hydroxylation reaction of a monophenol to o-diphenol leads to the oxidation in o-quinone. The proposed device concept aims to help retailers and consumers decide upon selling and buying according to the remaining shelf life, thus promoting sustainability related to food processes.

## 1. Introduction

Nowadays, the progressive population increase, as well as a situation of economic crisis and production deficit in several developed countries, heightens the need to reduce losses and waste of food to increase the world’s food intake. This is considered an ethical commitment to be assumed by us all, thus improving the nutritional level of the population and, consequently, fighting hunger [1]. To achieve this goal, an alliance is needed between the countries’ policies and their populations, as well as to motivate all the elements of the food chain, towards a modification of attitudes, applied processes, and effective management systems [2]. In addition, the food safety and quality in which the marketed products reach the end consumer is an increased concern [3]. As temperature is one of the factors that most influences the state of food preservation, there is a need to develop and test new appropriate sensors. Time temperature integrators (TTI), utilize the introduction of proteins or other chemical systems to quantify the impact of quality or safety of thermal processes and are one of the more promising options [3]. These sensors are one of the main missing links to improving the introduction of new monitoring technologies which will reduce production costs, predict food quality and safety, and may have a positive effect on mitigating food waste [2]. This need for monitoring is increased when the food products are highly perishable as they require stricter, qualified, and rigorous control [4]. The purpose of this paper is to contribute to the implementation of TTI in the commercialization of cherries, promote food quality and safety, reduce food waste, provide a way to predict the best time to sell the food products or predict the remaining shelf life, and thus foster the commercialization gains by reducing losses. Thus, this paper describes the concepts related to the formulation and design of an enzymatic time-temperature integrator device for the chromatic quality check of cherries. This will allow cherry quality to be monitored along the supply chain, from the trader to the consumer, through an interactive and visual device. That is, anywhere and anytime, human beings will be able to check whether the cherries are optimal or unfit for consumption. Moreover, using this enzymatic time-temperature integrator device, it will be possible to control and optimise the flows and times of the entire cherry supply chain and consecutively reduce waste. Likewise, as it is an enzymatic device, it can be explored and applied to other food products whose composition includes phenolic compounds and whose deterioration involves the degradation of these compounds.

### 1.1. Fruits and Vegetables: State of the Art

Fruits and vegetables are classified under agricultural products, but they stand out essentially for their perishability and the impact that their visual appearance has on the choice of the consumer at the moment of purchase. The particular characteristics of horticultural products (morphological, anatomical, physiological, and chemical) are responsible for the precocity of their degradation compared to other food products [5].

Besides being used for immediate consumption, fresh fruits and vegetables are important raw materials for many food industries, including wine, olive oil, juices, and frozen products. In most commercial establishments the quality and freshness of horticultural products can stand out positively [5]. 

Quality models have always been developed to predict the quality of different perishable products being subjected to different environments, in terms of the temperature and the atmospheric composition [6]. These factors are peculiarly important, not only when the products are arranged in large bulk storage rooms, but also because horticultural products generate heat throughout the storage process [7].

The main objective of the manufacturers is to offer safe products with the maximum possible nutritional value. The two techniques most used in the evaluation of thermal processes are the in-situ approach and the physical-mathematical method. In the in situ methods, changes in the product are monitored before and after processing to provide direct and accurate information about the food status. However, in practice assessing the microbial count, texture, vitamin content, and organoleptic quality are laborious, time-consuming, expensive, and in some cases, impossible due to the value detection limit of available techniques and/or sampling requirements [8].

#### Cova da Beira’s Cherries

Cherry tree cultivation occupies about 14% of the area of fresh fruits in Portugal and reaches 2191 ha in the Beira Interior region, constituting more than 50% of the national production of the cherry tree [9]. According to the systematic classification, the cherry tree belongs to the family *Rosaceae*, subfamily *Prunoideae*, genus *Pronus* L., and species *Prunus Avium* L. Sweet cherries, which are referred to throughout this document, come from regions with a suitable climate, found in the Mediterranean, Central Europe, North Africa, Near and Far East, South Australia, New Zealand, the United States of America, Canada, Argentina, and Chile and have a harvest period between May and July [10].

Cherries are very susceptible to mechanical damage as well as being highly perishable fruits presenting a very limited harvest period. This process requires a large number of tests to enable their storage time and preserve all their components. Unavoidably, the control and monitoring of this product are central and essential for optimal consumption in its highest state of quality, as well as for human health. Economically, it is also an advantageous process, as it translates into the reduction of food waste [10,11].

The characteristics most valued by the consumer are the dark red color, a sweetened flavor, a large diameter (about 28–30 mm), no cracks, and a perfectly rounded shape [11]. The quality of the fruit is often evaluated through its firmness since this directly affects the shelf life, consumer acceptance, and susceptibility not only to mechanical damage but also to infection by microorganisms [12,13].

Unsurprisingly, the climatic conditions, cultivation, maturation time, and subsequent harvesting and storage directly influence all these characteristics and define which of the cherries falls to the consumer’s choice [14].

##### Compounds in Cherries and Their Relevance

*Prunus avium* contains numerous chemical elements such as calcium, iron, proteins, fibers, organic acids, magnesium, minerals, potassium, phosphorus, and vitamins A, B, and C. Regarding the benefits of cherries’ consumption, they provide the medical field with a soothing, disinfecting, detoxifying, diuretic, intestinal regulator, antibacterial, and anti-inflammatory, among others. In addition, the consumption of cherries has shown benefits to alleviate gout and arthritis pain, as well as neurological, gastrointestinal, tumoral, and cardiovascular pathologies. These properties will only be recognized as beneficial if the cherry is in the perfect condition for consumption, otherwise it will not be able to exert the same effects [15,16]. Polyphenols directly influence color, sensory properties, and nutritional properties. To improve marketing, cherries as well as other food products, are processed in several ways. Before consumption, the functional and nutritional characteristics as well as their attributes and preservation capacity are improved [17]. 

In horticultural products, there is a vast diversity of phenolic compounds, the result of innumerable combinations of nature. Chemically, the phenolic compounds have a varied nomenclature due to the significant structural differences. They are classified according to the number of phenol rings as well as their binding. Of course, polyphenols have a common structure, which integrates an aromatic ring surrounded by one or more hydroxyl groups, passing through from simple phenolic molecules to molecules with a high degree of polymerization [15,18]. Phenolic compounds play an important role in the growth and reproduction of plants which not only gives them metabolic elasticity to the environmental alterations to which they are exposed but also affects their pigmentation. The bioavailability of polyphenols and their concentration in tissues varies depending on the structure [15]. Sweet cherries are rich in phenolic compounds, namely hydroxycinnamic acid derivatives such as neo-chlorogenic acid, p-coumaroyl quinic acid, and chlorogenic acid. These phenolic acids have been reported to be important for their potential contribution to the color of cherry fruits through co-pigmentation with anthocyanins [19]. These components are also related to general fruit maturation and the prevention of the enzymatic browning that happens through enzymatic reactions. The enzyme responsible for browning reactions is polyphenol oxidase, also known as PPO, which uses oxygen to catalyze the hydroxylation of phenols and their further oxidations to colored and highly reactive quinones. These last ones readily polymerize and/or react with endogenous amino acids and proteins to form complex brown pigments, which leads to organoleptic and nutritional modifications. Thus, these changes bring a serious problem in the food industry by depreciating the food value [20,21].

##### Methods of Conservation and Marketing Parameters

Horticultural products are living tissues, which undergo constant transformations during the period of harvest. Of these transformations, some are desirable for the consumer, but many of them are considered problematic and damaging to their quality, even culminating in loss [22]. 

The conservation process must be aimed at inhibiting microbial propagation while preserving the quality of the product [23]. Generally, it is common to resort to low temperatures to retard this growth or to high temperatures to delay growth. During dehydration, pH decreases, and other maintenance techniques use several chemical additives with preservative functions, which allow for the control of the surrounding environment to which the product is submitted [24]. In addition to temperature, the ideal conservation environment can also be achieved by handling the concentration of oxygen and carbon dioxide and by manipulating the relative humidity (to reduce water losses) through air circulation, since the temperature of the air goes up as heat transfers from the product [22]. In the cherry preservation process, which is susceptible to damage due to cold temperatures below 0 °C, the optimum storage temperature is around 1 °C [25]. The temperature for transport should be between 0 and 3 °C. The relative humidity should be between 90 and 95% and the concentrations of oxygen and carbon dioxide should be 0.5 to 2% and 20 to 25%, respectively [25]. The cherry, because of the yield of the harvest, has a relatively low impact on the total production of fresh fruits in Portugal. However, their yield is not affected by their market value, being a fruit that usually generates good revenue for the producers [9].

The export process should consider the fragility of the cherry, carefully adjusting the handling of this fruit. Thus, the success of this process is dependent on obtaining cherries with the highest possible quality, a good dimension, the adequacy of modern postharvest strategies, and the optimization of distribution and marketing circuits [24,26,27]. 

##### Microbial Contamination

During the post-harvest period, cracks and bruises are the most frequent damages in cherries and are a direct consequence of the rupture and collapse of the mesocarp cells, caused by the impact and compression of the fruits during development, harvesting, transport, and consequent handling [23,27].

Pathogenic fungi arising in the post-harvest period of the cherry are one of the main causes of losses in the fresh market. In sweet cherries, the main pathogenic microorganisms responsible for the alteration of the cherry quality parameters are *Penicillium expansum*, *Monilinia fructicola* (spp.), *Botrytis cinerea*, *Alternaria* spp., and *Rhizopus stolonifer* [23].

The *Monilinia fructicola* was first observed at the beginning of October 2015, in peaches imported from Italy and Spain. Visually an early brown spot (rot) was observed, which rapidly evolved and spread throughout the fruit in the form of conidia, which reproduce asexually, emerging as spores. Afterward, the studies carried out were able to isolate M11 and M13 genes, based on molecular morphological characteristics. Pathogenicity was tested by incubating mature peaches and sterilizing conidia. Five days after incubation, typical rot symptoms developed in incubated fruits, unlike control fruits which remained healthy [28]. *Moniliosis* can cause damage to the fruits after harvesting and during the storage period. Although the fruits are susceptible at all phases, the vulnerability reaches its peak at the beginning of maturation. In the presence of *Monilinia fruticula*, the fruits are completely rotten, covered with spores exhibiting the appearance of “creamy white” characteristics, and easily recognized in about five days [29]. In the case of the cherry, it can be infected by other cherries in direct contact or through spores transported by air, rain, or insects that penetrate the flowers. However, the main factors that contribute to cherry infections are the injuries caused by rain, hail, wind, cracks, and contact with insects and with other fruits. Again, favorable conditions such as humidity and mild temperatures provide fungal proliferation [23,29].

In addition, the most serious bacterial pathology in cherries is the bacterial canker caused by *Pseudomonas syringae*. In this case, it produces virulent polysaccharides that are fundamental for the creation of biofilms for bacterial survival and proliferation. Some of these polysaccharides are important in the initial phase of infection as they mask and protect the microbial agent from the host’s defense mechanisms. Other polysaccharides produced by *P. syringae* lead to hydrolysis reactions, consequent fructose release, and ripening [30].

### 1.2. Temperature Integrating Devices

To overcome the limitations and disadvantages inherent in these two methods, TTIs were developed as an alternative for the monitoring of these processes, especially perishable products [31]. A TTI can be defined as “a small measuring device which shows a measurable, temperature-dependent irreversible change, which easily and accurately illustrates the changes of a target attribute, being subject to temperature variation” [32]. The attribute of destination may be related to safety issues or, for example, inactivation of microorganisms (spores), or to the loss of a particular vitamin, texture, or color. The change during the thermal process must inevitably be irreversible to be able to quantify the impact of the process after processing. Exceptionally, after reading the monitoring system, there is the possibility of reversing the change to reuse the indicator [8]. The main advantage of TTIs is the ability to quantify the integrated time-temperature impact of the target attribute without any information about the real-time history and temperature of the product. The TTIs can be used as an alternative tool in the design, evaluation, and optimization of the processes when the approach of conventional methods is not viable [33].

#### Enzymatic TTI

Temperature variation over time can be easily monitored. When a reference is made to enzymatic systems, certain properties determine the kinetic parameters for thermal inactivation. The temperature sensitivity of the rate constant, *E_a_*, and the rate constant, *k*_TTI_, can be reported. Equation (1) gives the Arrhenius expression that illustrates the temperature dependence of the product quality [31]:

(1) $$\propto k_{{ref}_{}}\exp\left(\frac{{-E}_{a}}{R}\left(\frac{1}{T}-\frac{1}{T_{ref}}\right)\right)$$

where *k_ref_* is the kinetic constant of the reaction at a reference temperature *T_ref_*, *E_a_* is the activation energy (temperature sensitivity) of the reaction that controls the loss of quality, and *R* is the universal gas constant. Most of the enzymes used in the development of TTIs reveal relatively natural thermal stability. Temperature variation over time can be easily monitored [34]. Several types of TTIs can be highlighted, such as CheckPoint^®^ which is an adhesive label that reacts to time and temperature in a similar way to the food product. This process allows an evaluation of the qualitative parameters of the product under study since it reacts to the existing enzymatic activity [35]. This type of TTI is based on a physiological, visible principle; it is translated in a change of color, which is due to a decrease in the pH. This pH variation is a consequence of the controlled enzymatic hydrolysis of a given lipid substrate. The hydrolysis process will cause a release of an acid, which can be verified by the progressive alteration of the color, starting from a deep green and culminating in an orange-red [35,36]. Another commercial TTI is Fresh-Check^®^, which is based on a chemical polymerization reaction, where the colorless acetylene monomer polymerizes and becomes opaque. The temperature-dependent reaction occurs in the solid state, with the reaction product having a colored polymer as the monomer polymerizes [8]. This formation of the polymer, which is accompanied by the change in color, is explained by the decrease in reflectance. Since reflectance is the ratio between the flux of electromagnetic radiation incident on a surface and the reflected flux, there is a clear decrease in the latter [8]. 

The shape of the TTI is circular, whereby the color of the “active” center is evaluated and compared to the reference color of the surrounding ring, the color changes extending from the transparent to a black threshold. At a stage before the use of this TTI, care must be taken to store them frozen (at around −24 °C), since they are active during manufacture to improve the yield of the chemical reaction [7]. The Fresh Check^®^ indicator can be applied to a package in a self-adhesive format, such as a circle surrounded by a dark-colored ring [8,37]. Finally, OnVu™ can be considered an indicator of freshness, not replacing the expiration date, but providing a complement of information. The dynamics of operation are based on a solid-state photochromic reaction, which occurs due to the presence of organic crystals that alter the color according to the temperature variation registered [8]. The organic crystals present in the OnVu™ form the basis for the pigment that is used in the formation of the water paint, which enables the color change, and in this case is perceptible as a change from colorless to dark blue after irradiation with UV [8,9,10,11,12,13,14,15,16,17,18,19,20,21,22,23,24,25,26,27,28,29,30,31,32,33,34,35,36,37,38].

## 2. Materials and Methods

To develop this work and promote cherry quality and safety, while consecutively fostering commercialization gains by reducing losses, this paper describes the concepts related to the formulation and design of an enzymatic time-temperature integrator device for chromatic quality check of cherries. 

To perform this, firstly, the microbial kinetics of cherries were analyzed and deducted through ComBase. This was done to describe how microorganisms survive and proliferate, depending on certain predefined conditions (such as temperature, humidity, and pH), which directly influences the food [39]. ComBase Online Predictor is an online database of quantified microbial responses in diverse food environments that allows for predicting microbial behavior in food [40]. Thus, the microbial kinetics of the logarithmic function that translates bacterial growth as a function of time can be predicted. The main objectives are to verify when the infectious dose is reached, and to predict when the degradation process begins. Furthermore, the principal goal of this paper is to present a new design and formulation concept of an enzymatic time-temperature integrator device. The mechanism of application of the Cherry-TTI is described with the respective design and illustration.

## 3. Results and Discussion

Refrigeration is a preservation method which is considered necessary not only for cherries but also for all minimally processed fruits and vegetables. However, as with all beneficial processes, it also has its drawbacks, namely temperature abuse, which occurs in the time interval after packaging, during the subsequent distribution and transportation, ending with storage. To monitor the quality and condition of the cherry, it is proposed to create a specific TTI.

### 3.1. Microbial Kinetics: Deduction for the Specific Case of CHERRY

**Figure 1:**
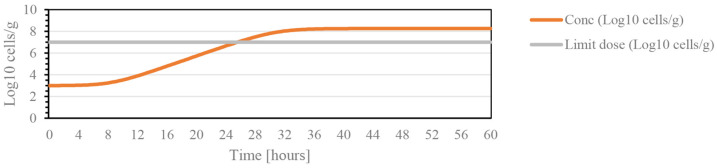
Graph illustrating the growth kinetics of the bacteria *Pseudomonas*, for a temperature of 20 °C, pH 7 (maximum value), with the initial dose of 3 log 10 cells/g during a period of 60 h.

In cherries, the most serious pathology is the bacterial canker caused by *Pseudomonas syringae*. Therefore, through the ComBase online tool, the microbial kinetics of this bacteria were predicted and the values of initial and infectious doses for several microorganisms are shown in Table 1. *T*_min_ is the minimum temperature for the beginning of the log phase; that is, the phase of microbial growth that leads to infectious development for the different microorganisms. As shown in Figure 1, the effective dose is reached substantially after 29 h, which indicates that from this moment, monitoring with greater acuity should be performed.

### 3.2. Concepts of the Formulation and Design of the TTI Prototype

**Figure 2:**
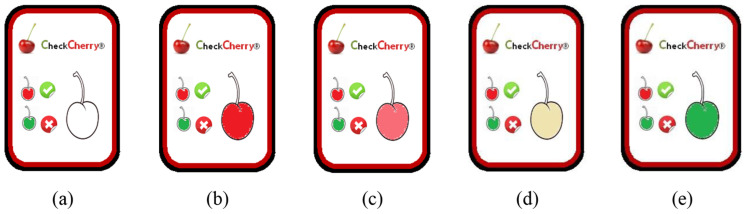
A scheme that translated the overall cherry quality development: (**a**) TTI present “not activated”, therefore not valid for evaluation; (**b**) indicates the ideal consumption status of the cherry—maximum quality detected; (**c**) provides a non-TTI visual indication that the cherry has entered the beginning of the degradation process—dates can be sent with maximum attention; (**d**) indicates an aggravated degradation process with mass degradation of the phenolic compounds; (**e**) provides an indication that cherry consumption is not recommended.

The use of TTI devices for cherries is particularly beneficial, not only for manufacturers but also for transport operators. However, the final consumer has an extremely important role and therefore has the right to be able to check what product conditions are about to be bought, by tracking the location and extent of exposure to adequate temperatures. A proposal concerning the concepts related to the formulation and design of a TTI for cherries is thus presented, as shown in Figure 2, named CheckCherry. When stored under suitable temperature conditions, the TTI indicator will keep the inside of the cherry red, the usual color of cherries.

The enzymes present in the TTI will change the color of the image as a function of the accumulation of the time-temperature history for the microorganisms described previously, considering the growth curves. This TTI is based on a physiological principle, visibility, that is, it translates into a color change caused by an oxidation reaction. With regards to the detected enzymatic process, degradation of the phenolic compounds occurs, which are substrates of the polyphenol oxidase enzyme, whose hydroxylation reaction of a monophenol in o-diphenol culminates in oxidation to o-quinone. The polyphenol oxidase enzyme catalyzes the oxidation of polyphenols in quinones that react non-enzymatically, producing colored pigments.

The fact that polyphenol oxidase and its substrates are present in different cell compartments means that enzymatic darkening is a direct consequence of tissue disintegration. The integrity of the membrane is lost after damage to the tissues during senescence or injury. This destroys the biological barrier between the polyphenol oxidase and its substrates leading to the rapid oxidation of phenols and the consequent production of dark pigments.

## 4. Conclusions

It was suggested that, based on the enzymatic reactions that occur, the enzymatic-type TTI would be the most appropriate to fit the specific case of Cova da Beira’s cherries. Phenolic compounds are thus the key, so that an enzymatic substrate can be detected indicating to the producer, distributor, and consumer, the actual state of conservation of the cherry, thereby leaving aside “random” predictions. Although many microorganisms affect the cherry, they are not easily detectable by the TTI, also because many other environmental conditions directly influence them, and it is not possible to predict with certainty that the detection by the labile substance of the TTI would be reliable. In short, the food waste and the safety of the cherry may be predicted by the use of TTIs, ensuring control of all phases of the cherry chain. The objective of promoting product profitability and investigating ways and procedures to provide as much information as possible in real-time is thus achieved while awaiting the future implementation of this technology in Portugal. In the future, the implementation of TTIs either in Portugal or worldwide should be a priority, not only for the case of Cova da Beira’s cherry but for all products. Priority should be given to TTIs for the most perishable products, such as meat, fish, all types of fruits and vegetables, and shellfish, and later for other products where the shelf life is often merely a suggestion or a strategy related to marketing. Intelligent packaging systems are considered an asset that helps predict the degradation of perishable products in general. However, more studies are needed to combine atmospheric components that destroy the development of certain microorganisms, but also not increase the proliferation of others. Finally, the combined use of TTIs with the new packaging will certainly be the future of trade, although costs need to be revised.

## Figures and Tables

**Table 1:** Limit dose values for pathogenic microorganisms.

Microorganism	*T*_min_ [°C]	Initial Colony [log 10 cells/g]	Infecting Colony [log 10 cells/g]
*Bacillus cereus*	5.0	3	>5
*Escherichia coli*	10.0	2	>6
*Listeria monocytogenes*	1.0	1.30	>2
*Staphylococcus aureus*	7.5	1.30	>5
*Salmonella*	7.0	2	>5
*Yersinia enterocolitica*	−1.0	2	>7
*Brochothrix thermosphacta*	0.0	2	>7
*Pseudomonas* spp.	0.0	2	>7

## Data Availability

Data is contained within the article.
